# Inherent growth advantage of (pre)malignant hepatocytes associated with nuclear translocation of pro-transforming growth factor *α*

**DOI:** 10.1038/sj.bjc.6602191

**Published:** 2004-11-09

**Authors:** E Schausberger, K Hufnagl, W Parzefall, C Gerner, D Kandioler-Eckersberger, F Wrba, M Klimpfinger, R Schulte-Hermann, B Grasl-Kraupp

**Affiliations:** 1 Institute for Cancer Research, Medical University of Vienna, Borschkegasse 8a, A-1090 Vienna, Austria; 2 University Hospital for Surgery, AKH-Vienna, Währinger Gürtel 18-20, A-1090 Vienna, Austria; 3 Institute for Clinical Pathology, AKH-Vienna, Währinger Gürtel 18-20, A-1090 Vienna, Austria; 4 Institute for Pathology and Bacteriology, Kaiser-Franz-Josef-Spital, Kundratstraße 3, A-1100 Vienna, Austria

**Keywords:** hepatocarcinogenesis, transforming growth factor *α*, pro-peptide, tumour prestages, growth regulation

## Abstract

The pro-peptide of transforming growth factor *α* (*proTGFα*) was recently found in hepatocyte nuclei preparing for DNA replication, which suggests a role of nuclear *proTGFα* for mitogenic signalling. This study investigates whether the nuclear occurrence of the pro-peptide is involved in the altered growth regulation of (pre)malignant hepatocytes. In human hepatocarcinogenesis, the incidence of *proTGFα*-positive and replicating nuclei gradually increased from normal liver, to dysplastic nodules, to hepatocellular carcinoma. *ProTGFα*-positive nuclei almost always were in DNA synthesis. Also, in rat hepatocarcinogenesis, *proTGFα*-positive nuclei occurred in (pre)malignant hepatocytes at significantly higher incidences than in unaltered hepatocytes. For functional studies unaltered (GSTp**^−^**) and premalignant (GSTp^+^) rat hepatocytes were isolated by collagenase perfusion and cultivated. Again, DNA synthesis occurred almost exclusively in *proTGFα*-positive nuclei. GSTp^+^ hepatocytes showed an ∼3-fold higher frequency of *proTGFα*-positive nuclei and DNA replication than GSTp**^−^** cells. Treatment of cultures with the mitogen cyproterone acetate (CPA) elevated the incidence of *proTGFα*-positive nuclei and DNA synthesis in parallel. Conversely, transforming growth factor *β*1 (TGF*β*1) lowered both. These effects of CPA and TGF*β*1 were significantly more pronounced in GSTp^+^ than in GSTp**^−^** hepatocytes. In conclusion, nuclear translocation of *proTGFα* increases in the course of hepatocarcinogenesis and appears to be involved in the inherent growth advantage of (pre)malignant hepatocytes.

Hepatocellular carcinoma (HCC) is one of the most common cancers worldwide, particularly in Asia and Africa, accounting for about 1 million deaths per year (Parkin *et al*, 2001). Recently, its incidence has substantially increased in Europe and the United States (El-Serag and Mason, 1999; Parkin *et al*, 2001). Transforming growth factor *α* (TGF*α*) is one of the cytokines, causally involved in the pathogenesis of liver cancer (Kiss *et al*, 1997; Grisham, 2001). It is produced by hepatocytes and nonparenchymal liver cells for paracrine, autocrine and/or juxtacrine stimulation, as shown in developing, regenerating, preneoplastic and neoplastic livers of rodents and humans (Mead and Fausto, 1989; Miller *et al*, 1995; Grisham, 1997; Kiss *et al*, 1997; Yarden and Sliwkowski, 2001). Transforming growth factor *α* appears to be upregulated in all stages of liver cancer development. Single hepatocytes, infected with the hepatitis-B virus, overexpress TGF*α* due to transactivation of the TGF*α* gene by the virus (Schirmacher *et al*, 1996). Hepatocellular adenomas and carcinomas as well as childhood hepatoblastomas have been found to be rich in TGF*α*, leading to elevated plasma levels of this cytokine (Yamaguchi *et al*, 1995; Kiss *et al*, 1997; Grisham, 2001).

According to textbook knowledge, TGF*α* is produced as a precursor transmembrane molecule (*proTGFα*). The ectodomain of the pro-peptide may be shed from the cell surface, where it may bind to and activate the erbb-1 receptor, that confers the growth signal via phosphorylation cascades to the nucleus (Massagué, 1990; Yarden and Sliwkowski, 2001). Considering the upregulation of TGF*α* in human malignancies, including liver cancer, hope focuses on the possible therapeutic benefit of blocking TGF*α*-evoked signal transduction on the cell surface, for example, by blockade of the receptor or of ligand–receptor interactions (Levitzki and Gazit, 1995; Mendelsohn, 1997). In a recent study, however, we have shown that hepatocytes in the intact liver and in primary culture synthesise *proTGFα* that translocates to the nucleus, where it appears to be involved in the mitogenic response of the cell (Grasl-Kraupp *et al*, 2002). This proposed novel pathway was induced by various different growth stimuli and is active in three different mammalian species, including humans. In mouse hepatocytes, almost all of the *proTGFα*-pos nuclei were also positive for erbb-1 (Schausberger *et al*, 2003). Moreover, several very recent papers suggest that the erbb receptors 1, 3 and 4 may bypass the protein phosphorylation cascades for transducing mitogenic stimuli (Lin *et al*, 2001; Ni *et al*, 2001; Offterdinger *et al*, 2002). Thus, there is considerable evidence of a direct action of growth factors/growth factor receptors from the EGF/erbb-receptor family in the nucleus (Wells and Marti, 2002). The question emerges whether the nuclear occurrence of *proTGFα* is involved in the altered growth regulation of (pre)malignant cells.

Rodent liver provides excellent tools for functional studies on hepatocarcinogenesis (Pitot, 1990; Grasl-Kraupp *et al*, 1997; Grisham, 1997). Treatment of rats with genotoxic carcinogens, such as *N*-nitrosomorpholine (NNM), induces single initiated hepatocytes that are detectable by their selective immunoreactivity for placental glutathione-S-transferase (GSTp^+^ cells); a considerable fraction of these cells develops to GSTp^+^ (pre)malignancy (Grasl-Kraupp *et al*, 2000). Although human HCC often do not express GSTp due to epigenetic silencing of the gene, GSTp^+^ lesions of rats and (pre)malignant lesions in human liver show significant similarities, such as mutations of the wnt-pathway, overexpression of TGF*α*, IGF-I and -II and other growth factors (Miller *et al*, 1995; Grisham, 1997; Yamada *et al*, 1999; Yang *et al*, 2003). Rates of replication and death of GSTp^+^ cells, and thus overall cell turnover, are somewhat reduced in the single-cell stage, but are significantly increased from the two-cell stage onwards (Grasl-Kraupp *et al*, 1997, 2000). Thus, initiation causes a change in the growth-regulatory network that becomes evident after the first replication cylce of GSTp^+^ cells. Treatment with tumour promoters, such as the progestin cyproterone acetate (CPA), or increased food intake further increases cell replication in preneoplasia, which is analogous to human hepatocarcinogenesis driven by anabolic steroids or overnutrition (Schulte-Hermann *et al*, 1990; Grasl-Kraupp *et al*, 1994; Fiel *et al*, 1996; Nair *et al*, 2002). This enhanced sensitivity of liver preneoplasia towards the various growth stimuli could result from an altered uptake, production and/or processing of endogeneous growth regulatory factors by the premalignant cell compartment.

In the present study, we asked whether the nuclear occurrence of *proTGF*á is involved (i) in the altered growth regulation of (pre)malignant hepatocytes and (ii) in the enhanced sensitivity of these cells towards induction of DNA replication by known growth stimulators. Recently, preneoplastic rat liver cells have become available for investigation in an *ex vivo* culture model (Löw-Baselli *et al*, 2000b). We applied this model, in combination with studies on human livers, and found that *proTGFα-*positive nuclei increased in the course of hepatocarcinogenesis. The possible role of nuclear *proTGFα* for the growth advantage of (pre)malignant cells is discussed with regard to tumour-therapeutic strategies targeted at TGF*α*/erbb-1 interactions on the cell surface.

## MATERIALS AND METHODS

### Human liver samples

Patients suffering from dysplastic liver nodules (*n*=9), hepatocellular adenoma (*n*=3), or HCC (*n*=10) were resected; chemotherapy had not been applied before surgery. Tissue samples were immediately fixed in 10% buffered formaldehyde. Classification of liver lesions and stage of disease followed published guidelines (Edmondson and Steiner, 1954; Hermank *et al*, 1993; International Working Party, 1995). For further details, see Table 1

**Table 1:** Age and sex of the patients, causative factors for the development of disease, histopathological diagnoses and TNM classification

**Age**	**Sex**	**Aetiology**	**Liver changes**	**Diagnosis**
27	f	Glycogenosis 1	S, glycogenosis	HCA
33	f		S	HCA
46	f			HCA
39	m		S, H	DN-LG
37	f		S, H	DN-LG
48	m		F	DN-LG
25	f		U	DN-LG
52	f		U	DN-HG
55	m	HCV	C	DN-HG
61	m	HCV	C	DN-HG
50	m	HCV	C	DN-HG
77	m		C	DN-HG
63	f			HCC/1, pT1, pN0, pM0
59	f			HCC/1, pT2, pN0, pM0
67	m		C	HCC/2, pT2, pN0, pM0
64	m	Ethanol	C	HCC/2, pT3, pN0, pM0
68	m			HCC/2, pT3, pN0, pM0
59	f	HCV	C	HCC/2, pT3, pNx, pM0
53	m	Ethanol	C	HCC/2, pT4, pN0, pM0
63	m	HCV		HCC/2, pT4, pN0, pM0
58	m	Ethanol	C	HCC/3, pT4, pNx, pM0
77	f			HCC/3, pT4, pNx, pMx

m, male; f, female; HCV, hepatitis-C-virus infection; C, cirrhosis, F, fibrosis, H, hepatitis; S, steatosis; U, unaltered liver parenchyma; HCA, hepatocellular adenoma; DN-LG, low-grade dysplastic nodule; DN-HG, high-grade dysplastic nodule; HCC, hepatocellular carcinoma. HCC/1–3 refers to the histopathological grading of the HCCs according to published criteria (Edmondson and Steiner, 1954). The TNM staging (tumour/node/metastasis) of the disease followed the UICC guidelines (Hermank *et al*, 1993).

. Informed consent was obtained from all patients.

### Human hepatocyte and hepatoma cell lines

The human hepatoma cell lines HepG2 (ATCC-No HB-8065), Hep 2B2.1–7. (ATCC-No HB-8064), and WRL 68 (ATCC-No CL-48) were obtained from the American Type Culture Collection (Rockville, MD, USA). The cells were maintained *in vitro* at 37°C and 5% CO_2_ in Dulbecco's minimum essential medium (DMEM) supplemented with 5% foetal calf serum, 100 U ml^−1^ penicillin and 100 *μ*g ml^−1^ streptomycin (all obtained from Gibco, Life Technologies Inc., Gaitherburg, MD, USA). Once per week cells were passaged at a seed density of 1 × 10^6^ cells per 25 cm^2^ plate.

### Animals and treatment

Male SPF Wistar rats, about 3 weeks old, were obtained from the Institut für Versuchstierkunde und Genetik (Himberg, Austria). Animals were kept under standardised conditions and were fed powder diet (Altromin 1321N, Altromin, Lage, FRG). At 3 weeks before treatment, animals were adapted to rhythmic feeding (from 0900 to 1400 h). This procedure synchronises DNA synthesis in the liver to a single peak per day (Grasl-Kraupp *et al*, 1994, 2000). After adaptation, animals were treated with a single dose of NNM (Sigma, St Louis, MO, USA; 250 mg per 10 ml phosphate-buffered saline per kg body weight). Phenobarbital (PB) was admixed to the powder diet and was fed to a subgroup of rats from day 4 to 17 months post-NNM. Concentrations of PB were adjusted every 14 days to provide a daily dose of 50 mg kg^−1^ body weight (Löw-Baselli *et al*, 2000b). Animals were killed by decapitation under CO_2_ asphyxation. For further details, see Grasl-Kraupp *et al* (1994, 2000) and Löw-Baselli *et al* (2000a). All experiments were performed according to the ‘Austrian Guidelines for Animal Care and Protection’, which meet the standards required by the ‘UKCCCR Guidelines for the Welfare of Animals in Experimental Neoplasia’ (2nd edn.) (Workman *et al*, 1998).

### Histology

Human and rat liver samples, fixed in 10% buffered formaldehyde, were processed as described (Grasl-Kraupp *et al*, 1994, 2000; Löw-Baselli *et al*, 2000a); two serial sections, 1 *μ*m thick, were cut; one of the sections was stained for GSTp (rat only) or Ki-67 (human only), the second one was stained for TGF*α*.

#### Immunostaining for TGF*α*, GSTp and Ki-67

The primary antibodies used were rabbit polyclonal IgG against rat Yp-subunit of GSTp (Biotrin International, Dublin, Eire); mouse monoclonal IgG against recombinant mature TGF*α* encompassing amino acids 39–88 (clone 213-4.4, Oncogene Science, Uniondale, NY, USA); mouse monoclonal IgG against a synthetic peptide encompassing amino acids 144–160 of the C-terminus of *proTGFα* (Ab-3; InnoGenex, San Ramon, CA, USA); mouse monoclonal antibody against full-length recombinant Ki-67 protein (Dianova, Hamburg, FRG).

For TGF*α* or Ki-67 staining, formaldehyde-fixed and de-waxed tissue sections were placed in a glass Coplin jar filled with 0.01 M sodium citrate buffer, pH 6.0. Slides were heated for periods of 2 min at a maximal power setting (about 800 W) and for 2 × 2 min at the submaximal power setting (600 W). The citrate buffer reached boiling point within 2 min and the fluid level in the Coplin jar was topped up with distilled water between heating periods to prevent drying of the sections.

The following staining schedule was used: hydrogen peroxide to block endogenous peroxidases (3%, 20 min, room temperature); incubation with 2.5% bovine serum albumin (BSA) in TBS (0.05 M Tris, 0.3 M NaCl, pH 7.6; 30 min, room temperature); primary antibodies were diluted in 1% BSA-TBS (anti-Yp: 1 : 5000; anti-TGF*α*: 1 : 50; anti-Ki-67 : 1 : 50) and applied overnight at 4°C; rinsing with TBS; secondary antibodies were diluted in 2.5% BSA-TBS (biotinylated goat-anti-rabbit IgG or biotinylated rabbit-anti-mouse IgG; both 1 : 600, Dakopatts, Glostrup, Denmark) and were applied for 90 min at room temperature; rinsing with TBS was followed by incubation with streptavidin–horseradish peroxidase conjugates (1 : 300 in TBS, 45 min, room temperature; Dakopatts); diaminobenzidine (Sigma, St Louis, MO, USA) was used for colour development. The specificity of immunohistochemistry was confirmed by omitting the primary antibodies.

### Determination of DNA synthesis

#### Rat liver

^3^H-thymidine (6.7 Ci mmol^−1^; NEN, Frankfurt, FRG) was injected into the peritoneal cavity as a single dose of 0.2 mCi kg^−1^ body weight at the daily peak of DNA synthesis between 2000 and 2100 h (see above). After 36 h, animals were killed. GSTp-stained sections were coated with a solution of 1% gelatine (BioRad, Richmond, CA, USA) and 0.05% chromalaun (Merck, Darmstadt, FRG) and were air-dried. After autoradiography, the percentage of hepatocyte nuclei in DNA synthesis was determined for at least 1000 nuclei of unaltered cells in each liver and in all nucleated cells within individual GSTp^+^ foci (labelling index (LI)). Since interindividual variations were small, LIs obtained from different livers or foci of the same experimental group were pooled.

#### Human liver

In order to identify human hepatocyte nuclei in the S-phase of the cell cycle, serial sections were stained for TGF*α* and Ki-67 (see above). Individual Ki-67-positive nuclei were followed in the consecutive TGF*α*-stained serial section by overlaying the two images in two microscopes linked by a bridge for overprojection (Zeiss, Germany).

### Primary hepatocytes

Male SPF Wistar rats were obtained from the animal facilities of the Medical University of Vienna at the age of 3–4 weeks and were treated with a single dose of NNM (250 mg kg^−1^ body weight), as described above. After 21 days, livers were perfused with collagenase as described (Parzefall *et al*, 1989; Löw-Baselli *et al*, 2000b).

#### Treatment of primary hepatocyte cultures

Cells were seeded and kept under serum-free conditions, as described (Parzefall *et al*, 1989; Löw-Baselli *et al*, 2000b). Treatment commenced 4 h after plating (time point 0). A stock of 10 *μ*g (10 *μ*l)^−1^ of 10 mM acetic acid of human recombinant mature TGF*α* (UBI, Lake Placid, NY, USA) was prepared and was added to the medium for a final concentration of 10 ng ml^−1^. Tyrphostin A25 (synonym: tyrphostin AG82; Calbiochem, La Jolla, CA, USA) was dissolved in dimethylsulphoxide (DMSO) to obtain a stock of 10 mg ml^−1^; 1 *μ*l of this stock was added per ml medium. Cyproterone acetate, a gift from Schering AG (Berlin, FRG) was dissolved in DMSO. In all experiments, the final concentration of CPA was 10 *μ*mol in 0.2% solvent. Recombinant mature TGF*β*1 synthesised by CHO transfectants was supplied gratuitously by Bristol-Myers Squibb (Seattle, WA, USA). TGF*β*1 was dissolved as described (Oberhammer *et al*, 1992).

#### Double immunostaining of culture plates for GSTp and TGF*α*

Cells in culture were fixed for 90 min at room temperature with 4% buffered formalin according to Lillie and were then kept in distilled water at 4°C until immunostaining. Then, the following schedule was used: hydrogen peroxide to block endogenous peroxidases (3%, 20 min, room temperature); primary antibodies were diluted in 2.5% BSA in TBS (0.05 M Tris, 0.3 M NaCl, pH 7.6) (rabbit-anti-Yp 1 : 5000; mouse-anti-TGF*α* 1 : 50; mouse-anti-*proTGFα* 1 : 50) and applied overnight at 4°C; rinsing with TBS; secondary antibodies were diluted in 2.5% BSA–TBS (biotinylated goat-anti-mouse IgG or alkaline-phosphatase-labelled goat-anti-rabbit; both 1 : 600; Dakopatts) and were used for 90 min at room temperature; rinsing with TBS was followed by incubation with streptavidin (1 : 300 in TBS, 45 min, room temperature; Dakopatts); diaminobenzidine (Sigma), 5-bromo-4-chloro-3-indolylphosphate and nitro blue tetrazolium chloride (Boehringer, Mannheim, FRG) were used for colour development. Omission of the primary antibodies served as control.

#### Determination of DNA replication in cultured hepatocytes

Immunohistochemically stained plates were coated with 1% gelatine/0.05% chromalaun, air-dried, dipped into photo-emulsion (Ilford K5, Dreieich, FRG), and exposed for about 14 h. The plates were processed with a photographical developer and fixative, and were finally dried at room temperature and mounted in Kayser's glycerine gelatine (Merck, Darmstadt, FRG). The LI was calculated as percentage of labelled hepatocyte nuclei per total number of hepatocyte nuclei counted.

### Two-dimensional gel electrophoresis, immunoblotting and identification of protein spots

Nuclei and cytoplasm (postnuclear supernatant) of human hepatoma cells and of rat liver were separated according to the method of Tata, applying 2.0 M sucrose for purification (Tata, 1974). This was followed by a nuclear matrix preparation, as has been described in detail (Gerner *et al*, 1998).

The electrophoretically separated proteins were transferred onto PVDF sheets; the filters were soaked in excess blocking buffer ((3% BSA, Sigma, St Louis, MO, USA) in TBST buffer (10 mM Tris–HCl, pH 8, 150 mM NaCl, 0.1% Tween 20)). The mouse monoclonal antisera against amino acids 39–88 of mature TGF*α* (Oncogene Science, Ab-1, clone 134A-2B3; 1 : 300) were diluted in TBST buffer and were incubated overnight at 4°C. Thereafter, sheets were incubated for 1 h at room temperature with an alkaline phosphatase-conjugated anti-mouse IgG (Promega, Madison, WI, USA) diluted 1 : 7000 in TBST buffer containing 0.25% BSA; 5-bromo-4-chloro-3-indolylphosphate and nitro blue tetrazolium chloride (Boehringer, Mannheim, FRG) were used for staining.

For mass spectrometry fingerprinting, Coomassie Blue-stained proteins were directly cut out of preparative gels. Matrix-assisted laser desorption ionisation-time-of-flight (maldi-tof) of tryptic protein hydrolysates and protein identification were carried out essentially as described (Fountoulakis and Langen, 1997; Grasl-Kraupp *et al*, 2002). Proteins were considered identified by means of mass spectrometry fingerprinting when at least 15% of the whole sequence gave hits and when the molecular mass/pI values were identical to the ones calculated or published in 2D databases.

### Statistics

If not stated otherwise, data of at least three animals per time point and treatment group are given. Where indicated, the significance of differences of means was calculated by Kruskal–Wallis test or Wilcoxon test. For incidences, confidence intervals were calculated for *P*<0.05.

## RESULTS

### The pro-peptide of TGF*α* is present in the nuclei of human and rat hepatocytes and human hepatoma cell lines

**Figure 1:**
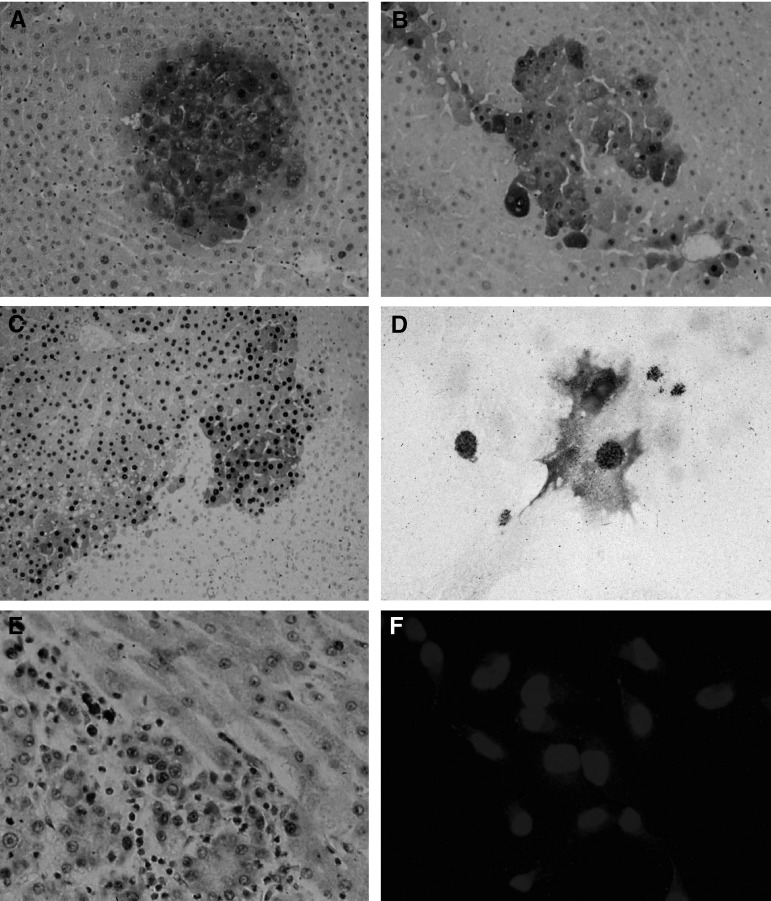
Occurrence of nuclear proTGF*α* in human and rat hepatocarcinogenesis. (**A–C**) Rat liver 12 months post-NNM: serial sections of a preneoplastic lesion stained for GSTp (**A**) and TGF*α* (**B**); (**C**) HCC with *proTGFα*-pos nuclei (**C**). (**D**) Preneoplastic GSTp^+^ hepatocytes (violet) with *proTGFα-*pos nucleus (brown) and incorporated ^3^H-thymidine (black spots) in primary culture; hepatocytes were isolated at day 21 post-NNM and cultured for 48 h; ^3^H-thymidine was added to the medium 24 h before harvesting. (**E**) Human HCC and (**F**) human HepG2-hepatoma cells with *proTGFα*-pos nuclei. Magnifications: × 50 for (**A**), (**B**), × 75 for (**E**); × 25 for (**C**); × 200 for (**D**, **F**).

**Figure 2:**
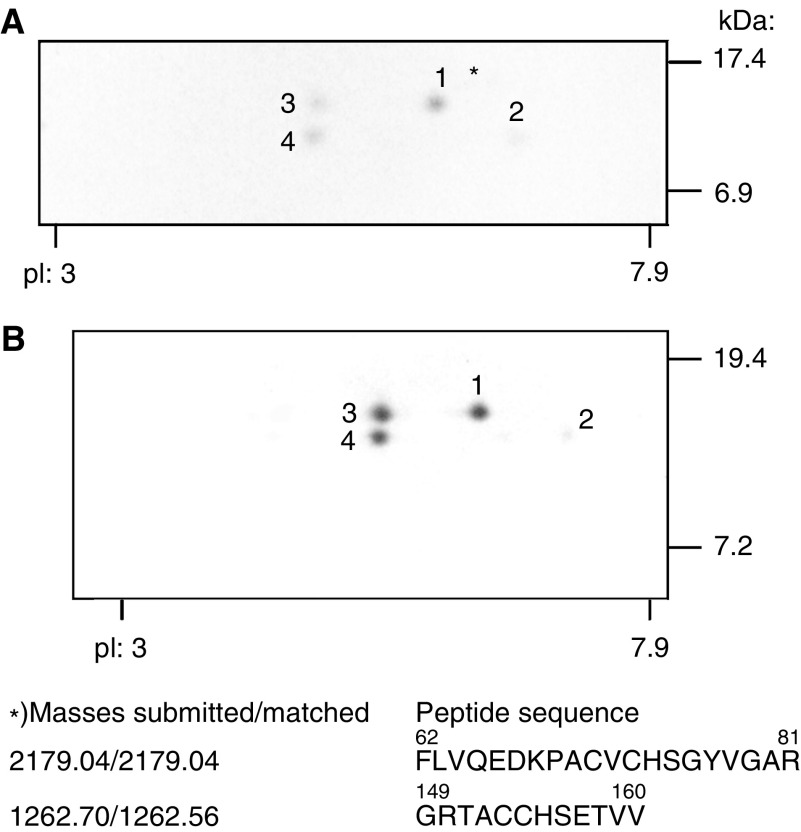
Detection of proTGF*α* in the nuclear fraction of human HepG2-hepatoma cells and of rat liver. Nuclear matrix proteins of hepatoma cells (**A**) and of an untreated male rat liver (**B**) were subjected to two-dimensional immunoblotting. The gels covered a range of pI 3–8 and Mr 4–40 kDa. (**A**) Spots 1–4 were analysed by maldi-tof: spot 1 was identified as wild-type *proTGFα*; ^*^ indicates the amino-acid sequence identified, which covers 19% of the total pro-peptide of TGF*α*.

In anti-TGF*α*-stained liver sections, immunoreaction was found within the cytoplasm, cell membranes and, most prominent, the nuclei of hepatocytes (Figure 1B, C and E

). Likewise, the nuclei of the hepatoma cell lines HepG2 (Figure 1F), WRL68 and Hep2B (not shown) displayed a strong immunoreaction. Nuclear matrices, prepared from human HepG2 cells (Figure 2A

) and from rat liver (Figure 2B), were separated by two-dimensional gel electrophoresis and were subsequently subjected to anti-TGF*α* immunoblotting; four spots around 17 kDa and an isoelectric point of 7.5 appeared. Spots 1–4 of HepG2 cells were subjected to maldi-tof analysis: spot 1 was verified to be the wild-type form of *proTGFα*; spot 2 was recently found to be wild-type *proTGFα* as well (Grasl-Kraupp *et al*, 2002). Spots 3 and 4 could not yet be identified. Immunostaining with antisera against the C-terminus of the pro-peptide also confirmed the presence of *proTGFα* in hepatocyte nuclei (see below). The mature form of TGF*α* at about 5.6 kDa was not detected.

### Occurrence of DNA replication and nuclear *proTGFα* in the different stages of human hepatocarcinogenesis

**Figure 3:**
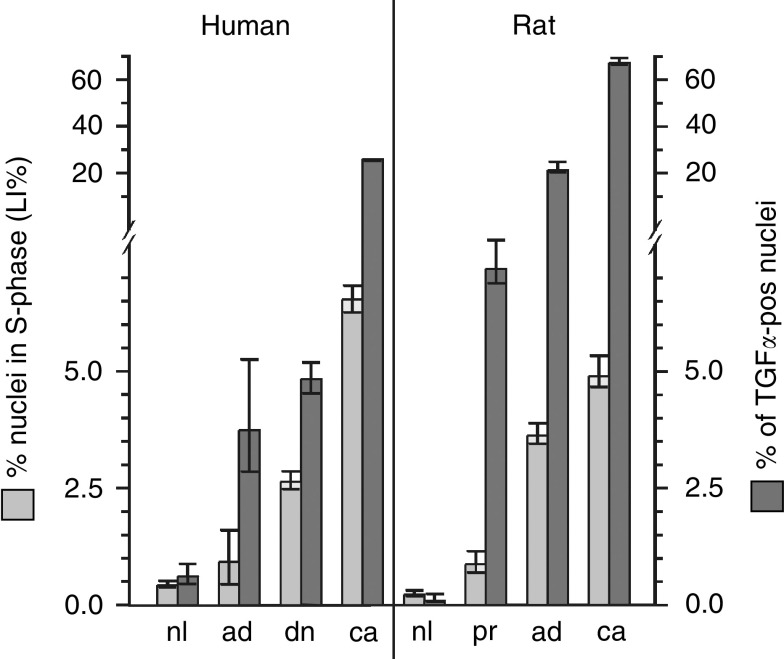
Increased cell replication and incidences of proTGF*α*-pos nuclei in human and rat hepatocarcinogenesis. White columns represent the percentage of nuclei in S-phase (LI%), dark columns the percentage of *proTGFα-*pos nuclei. Humans: three hepatocellular adenomas, nine dysplastic nodules, 10 heptocellular carcinomas and the surrounding liver of the 22 cases were evaluated. At least 1000 hepatocyte nuclei (on average 2055 nuclei) per liver or tumour were screened for Ki-67 and *proTGFα* positivity. Rats: time interval between ^3^H-thymidine injection and killing was 36 h. The percentages of *proTGFα-pos* nuclei and LI were determined at least in: 2000 nuclei of GSTp**^−^** and 1221 nuclei of GSTp^+^ hepatocytes per liver (*n*=13); 1381 nuclei per adenoma (*n*=15); 1362 nuclei per carcinoma (*n*=14). nl, normal liver; pr, preneoplastic GSTp^+^ lesion; ad, hepatocellular adenoma; dn, dysplastic nodule; ca, HCC. Incidences are given; vertical lines give 95% confidence intervals: no overlap indicates a statistically significant difference for *P*<0.05.

The percentage of both, replicating nuclei and *proTGFα*-pos nuclei, was gradually increased from normal liver, to hepatocellular adenoma, to dysplastic nodules, to HCC (Table 1, Figure 3

). In unaltered liver, replicating nuclei and *proTGFα-*pos nuclei occurred at a similar frequency. Premalignant and malignant liver lesions, however, revealed a much higher percentage of *proTGFα*-pos nuclei when compared to nuclei in S-phase (Figure 1E and 3). Staining for Ki-67 was used to identify hepatocyte nuclei in the S-phase of the cell cycle (for details, see Materials and methods); five HCC were studied: 77.3±9.2% of 343 Ki-67-positive hepatocyte nuclei evaluated displayed *proTGFα*. A high co-incidence of DNA synthesis and *proTGFα* was recently shown also for hepatocyte nuclei in cirrhotic liver (Grasl-Kraupp *et al*, 2002). Taken together, these findings indicate that a hepatocyte nucleus undergoing DNA replication almost always contains *proTGFα*. Further expression of *proTGFα* in nuclei not immediately undergoing DNA synthesis occurs predominantly in premalignant and malignant hepatocytes.

### Occurrence of DNA replication and nuclear *proTGFα* in the different stages of rat hepatocarcinogenesis

Hepatocarcinogenesis was induced in rats by application of NNM. This leads to the formation of single GSTp^+^ cells and small preneoplastic GSTp^+^ lesions, followed by expansive growth of some of the lesions. To enhance the formation of liver tumours, rats were treated with the tumour promoter PB (Löw-Baselli *et al*, 2000b). Analogous to the findings in human liver, *proTGFα*-positive nuclei occurred in (pre)malignant hepatocytes at a significantly higher incidence than in unaltered hepatocytes (Figures 1A–C and 3). This substantiates that the experimental model fits the human situation.

### Inherent growth advantage of cultured preneoplastic GSTp^+^ rat hepatocytes associated with nuclear *proTGFα*

To study the functional significance of nuclear *proTGFα* for hepatocarcinogenesis, unaltered GSTp**^−^** and preneoplastic GSTp^+^ cells were isolated from the livers by collagenase perfusion and cultivated. Cell isolation was performed on day 21 post-NNM treatment (no PB promotion). At this time point, there is the maximal occurrence of GSTp^+^ cell clones in the liver. Based on stereological calculations of the size distribution in the third dimension, only 23±11% of the isolated GSTp^+^ cells in culture derive from single-cell clones and 77±29% derive from small foci (three cells on average), with an inherently elevated cell turnover (DeGunst and Luebeck, 1998; Grasl-Kraupp *et al*, 2000).

**Figure 4:**
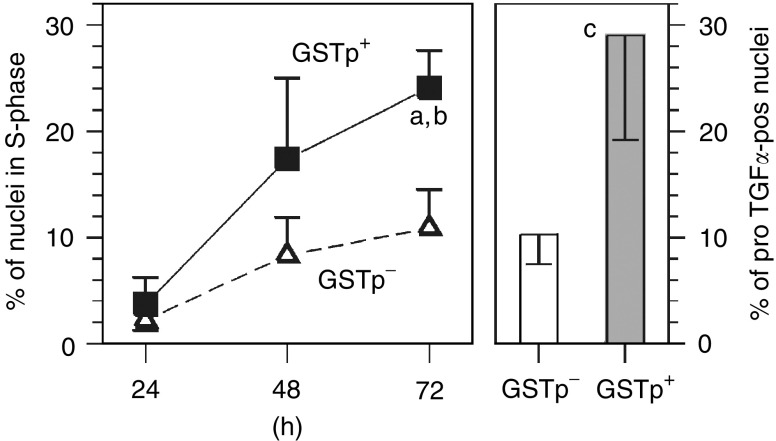
DNA synthesis (LI(%)) and % of proTGF*α*-pos nuclei in cultured GSTp**^−^** and GSTp^+^ hepatocytes. ^3^H-thymidine was added 24 h before harvesting of cells. The % of *proTGFα*-pos nuclei was determined after 48 h of culture. Symbols: Δ or open columns, GSTp**^−^** cells; ▪ or dark columns, GSTp^+^ cells. In each of the experiments, 2000 nuclei of GSTp**^−^** cells and 600 nuclei of GSTp^+^ cells were evaluated. Means±s.d. are given from separate experiments with cultures from five rats. Statistics of LI(%) in GSTp**^−^** *vs* GSTp^+^ cells over time-course by Kruskal–Wallis test: (a) *P*<0.001. Statistics of LI(%) in GSTp**^−^** cells *vs* GSTp^+^ cells at the last time point of evaluation by Wilcoxon's test: (b) *P*<0.01. Statistics of % of *proTGFα*-pos nuclei in GSTp**^−^** *vs* GSTp^+^ cells by Wilcoxon's test: (c) *P*<0.001.

Replicative DNA synthesis was generally high in the isolated hepatocytes (Figure 4

); this may be explained by the fact that the livers still underwent regeneration, as observed *in vivo* (Grasl-Kraupp *et al*, 2002). *ProTGFα* was present in the nuclei of about 10% of the hepatocytes in primary culture (Figure 4 and Table 2

**Table 2:** DNA synthesis occurs preferentially in *proTGFα-pos* nuclei

	**CO**		**CPA**		**TGF*β*1 (10 ng)**	
	**In GSTp^—^**	**In GSTp^+^**	**In GSTp^—^**	**In GSTp^+^**	**In GSTp^—^**	**In GSTp^+^**
% of *proTGFα-pos* nuclei	10.3±2.9	29.0±9.9^*^	20.7±3.2^**^	46.1±7.2^*^	2.0±1.1^**^	10.1±1.9^***,**^
% of *proTGFα-pos* nuclei in S phase	76.5±6.8	62.1±7.8	81.7±4.3	75.1±5.8	41.1±20.4	10.2±5.9^**^
% of *proTGFα-neg* nuclei	89.7±2.9	71.0±9.9^****^	79.3±3.2	53.9±7.2^***,****^	98.0±1.1^*****^	89.9±3.9^****,*****^
% of *proTGFα-neg* nuclei in S phase	4.5±1.3	6.9±4.9	9.6±3.6	12.5±5.7	1.0±1.0^*****^	0.6±0.4^*****^

^3^H-thymidine was added 24 h before harvesting of cells. The percentage of nuclei in S phase and of *proTGFα*-pos nuclei were determined after 48 h of culture. In each experiment, 2000 nuclei of GSTp^—^ cells and 600 nuclei of GSTp^+^ cells were evaluated. Means±s.d. are given from separate experiments with cultures from five rats. Statistics by Wilcoxon's test for GSTp^—^ cells *vs* GSTp^+^ cells. ^****^P<0.05, ^***^P<0.01, ^*^P<0.001; for Co *vs* treated group: ^*****^P<0.05, ^**^P<0.01.

). In any case, DNA replication occurred preferentially in *proTGFα*-pos nuclei in both GSTp**^−^** and GSTp^+^ hepatocytes (Table 2). This was also confirmed by immunostaining of parallel culture plates with two different antisera: DNA was synthesised by 76.5±6.8% of the nuclei being positive for amino acids 39–88 of *proTGFα* (encompassing the mature form) and by 65.3±19.2% of the nuclei being positive for amino acids 144–160 of the C-terminus of the pro-peptide. In both stains, DNA synthesis in negative nuclei was a rare event (see also Table 2).

GSTp^+^ cells showed an about three-fold higher frequency of *proTGFα*-pos nuclei and DNA replication than GSTp**^−^** cells (Figure 4 and Table 2). Thus, the inherent growth advantage of the cultured preneoplastic cell population appears to be highly associated with the enhanced nuclear translocation of *proTGFα*.

### Different signal transduction pathways of *TGFα* gene products in GSTp**^−^** and GSTp^+^ hepatocytes

**Figure 5:**
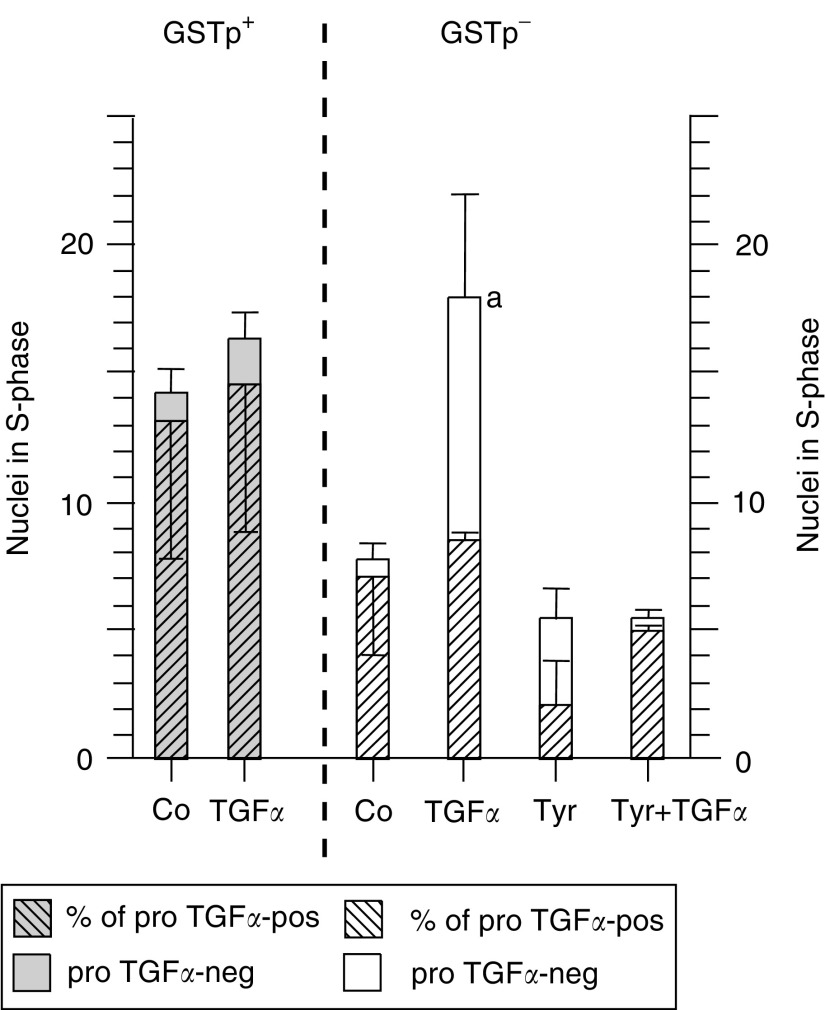
Mature TGF*α* increases the percentage of nuclei in S-phase (LI%) preferentially in GSTp**^−^** hepatocytes without nuclear proTGF*α*. ^3^H-thymidine was added 24 h before harvesting of cells. The percentages of replicating and of proTGF*α*-pos nuclei were determined after 48 h of culture. For the number of experiments and cells scored, see Figure 4. Hatched portions of the bars indicate LI of proTGF*α*-pos nuclei; nonhatched portions of the bars indicate LI of proTGF*α*-neg nuclei; the sum of the hatched and unhatched portion (total bar) indicates LI of all nuclei; Co: DMSO-control; TGF*α*: mature TGF*α*; Tyr: Tyrphostin A25. Statistics by Student's *t*-test: (a) *P*<0.05.

We have confirmed recently that in our system mature TGF*α* acts via erbb-1 in the cellular membrane of *proTGFα-*neg cells according to classical concepts of growth signal transduction (Grasl-Kraupp *et al*, 2002). Accordingly, in the present study, addition of mature TGF*α* increased DNA synthesis almost exclusively in the GSTp**^−^** cells that do not express *proTGFα.* This increase in DNA synthesis was blocked by the erbb-1-tyrosine kinase inhibitor tyrphostin A25 (Figure 5

). However, mature TGF*α* exerted no significant effect on any cell with nuclear *proTGFα*, which was most evident for the *proTGFα*-rich GSTp^+^ population (Figure 5). At present, it is unclear why TGF*α* exerted no effect on the GSTp^+^ cells with *proTGFα*-neg nuclei. The expression of yet unidentified growth factors may confer autonomy from exogeneous growth stimuli to this subpopulation of premalignant cells. Taken together, these findings suggest that two different TGF*α*-mediated signal transduction pathways are operative in different cell populations: the ‘classical’ erbb-1-mediated pathway of mature TGF*α* becomes active mostly in the *proTGFα*-poor GSTp**^−^** hepatocytes, while the second one, triggered by nuclear *proTGFα*, is effective in the *proTGFα*-rich preneoplastic GSTp^+^ cells.

### Transforming growth factor-*β*1 reduces DNA synthesis and nuclear proTGF*α* in cultured GSTp**^−^** and GSTp^+^ hepatocytes

**Figure 6:**
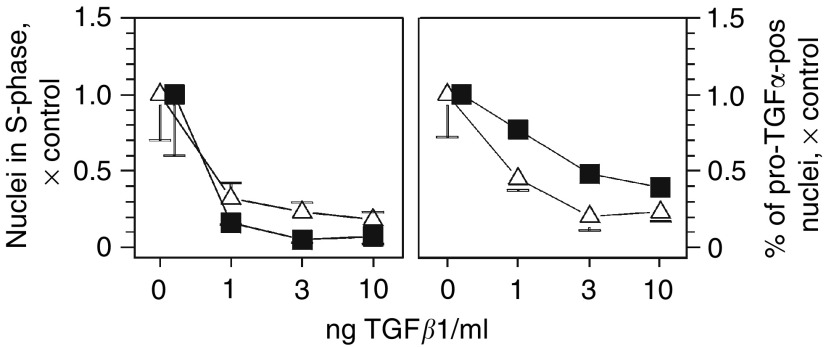
Transforming growth factor *β*1 reduces DNA synthesis and proTGF*α*-pos nuclei in GSTp**^−^** and GSTp^+^ cells cultured for 48 h. ^3^H-thymidine was added 24 h before harvesting of cells. Symbols: Δ, GSTp**^−^** cells, ▪, GSTp^+^ cells. Data are calculated as fold control; absolute values are given in Table 1; number of experiments and cells scored see Figure 4. Means±s.d. are given. Statistics by Kruskal–Wallis test for dose–response effects within a cell population: (a) *P*<0.01; (b) *P*<0.05.

DNA replication was suppressed by 1 ng of TGF*β*1 and even more by 3 ng of TGF*β*1 ml^−1^ medium in GSTp**^−^** and GSTp^+^ cells (Figure 6

). DNA replication was inhibited at the most effective concentration of the cytokine in about 66% of the GSTp**^−^** cells, but in at least 90% of the GSTp^+^ cells. These data suggest that TGF*β*1 acts more strongly on GSTp^+^ than on GSTp**^−^** cells. Furthermore, TGF*β*1 diminished the fraction of hepatocytes expressing nuclear *proTGFα* and synthesising DNA (Figure 6 and Table 2). Apparently, the suppression of DNA synthesis by TGF*β*1 in primary hepatocytes involves downregulation of nuclear *proTGFα*. This effect was evident for both, GSTp**^−^** and GSTp^+^ cells.

### The hepatomitogen CPA induces DNA synthesis and nuclear proTGF*α* in GSTp**^−^** and GSTp^+^ hepatocytes

**Figure 7:**
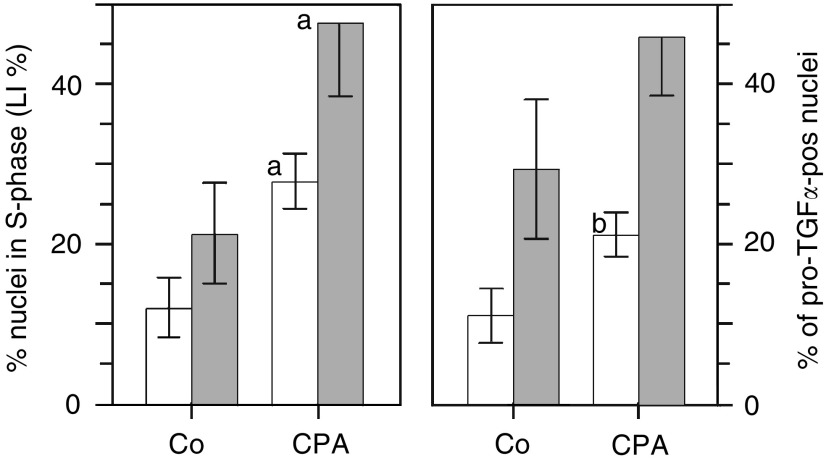
Cyproterone acetate induces DNA synthesis and proTGF*α*-pos nuclei in GSTp**^−^** and GSTp^+^ cells cultured for 48 h. ^3^H-thymidine was added 24 h before harvesting of cells. Symbols: light columns, GSTp**^−^** cells; dark columns, GSTp^+^ cells. For the number of experiments and cells scored, see Figure 4. Means±s.d. are given. Statistics by Wilcoxon's test for Co *vs* CPA: (a) *P*<0.01; (b) *P*<0.05.

Treatment with the hepatomitogen CPA doubled the LI in cultured GSTp**^−^** and GSTp^+^ cells (Figure 7

). Thus, every fifth GSTp^+^ cell was stimulated to DNA synthesis by CPA, while only every tenth GSTp**^−^** cell was recruited to the pool of replicating hepatocytes. We asked whether nuclear *proTGFα* might be involved in this enhanced growth stimulation of the preneoplastic cell population. In fact, the CPA-induced increases in DNA synthesis were closely paralleled by an equal elevation of *proTGFα*-pos nuclei (Figure7 and Table 2). This may indicate that the enhanced occurrence of nuclear *proTGFα* in liver preneoplasia may commit the preneoplastic cells for preferential response towards growth stimulation.

## DISCUSSION

The present paper describes that the pro-form of TGF*α* occurs in the nucleus of (pre)malignant rat and human hepatocytes, while mature TGF*α* was not detected. The translocation of *proTGFα* to the nucleus may contribute to the inherent growth advantage of early and late stages of hepatocarcinogenesis. The present observations extend recent findings on normal hepatocytes from rat, mouse and humans, which revealed that the translocation of *proTGFα* to the nucleus in G1 of the cell cycle is almost always followed by replicative DNA synthesis (Grasl-Kraupp *et al*, 2002). Nuclear translocation of *proTGFα* is induced by various hepatomitogenic stimuli, such as regenerative growth after partial hepatectomy or intoxication with CCl_4_ and hyperplastic growth induced by CPA in the intact animal. In culture, the incidence of *proTGFα-pos* hepatocyte nuclei is elevated by treatment with hepatomitogenic CPA, prostaglandins E2 and F2*α*, and hepatocyte growth factor (Grasl-Kraupp *et al*, 2002; Schausberger *et al*, 2003). Thus, a great variety of growth stimuli all involve nuclear translocation of *proTGFα*. This peptide may therefore serve as a kind of intracellular shortcut in mediating autocrine growth stimulation of normal, premalignant and malignant liver cells.

The possible interactions of *proTGFα* with the growth-regulatory machinery in the nucleus of unaltered and (pre)neoplastic hepatocytes are still unclear. Soluble or membrane-bound precursors of TGF*α* are biologically active, suggesting that the pro-form may attach to the binding site of erbb-1 as known for the mature form (Ignotz *et al*, 1986). We found that *proTGFα* and erbb-1 almost always co-localise within the nucleus of mouse hepatocytes, as shown by confocal laser-scanning microscopy (Schausberger *et al*, 2003). It is currently under investigation by FRET technology whether the large TGF*α* precursor attaches to erbb-1 and may be co-targeted to the nucleus as a receptor-bound ligand. This may provide the clue for the function of this pro-peptide for DNA synthesis, considering that erbb-1 may act as a transcription factor for cyclin D1 (Lin *et al*, 2001). Our data also suggest that the activity of *proTGFα* in the nucleus does not depend on an erbb-1 receptor tyrosine kinase activity, since DNA replication of *proTGFα*-pos nuclei was not affected by the tyrosine kinase inhibitor tyrphostin (Figure 5).

The cell culture system used in the present study allows to investigate the functional significance of nuclear *proTGFα* in premalignant cells; cultured GSTp^+^ hepatocytes showed significantly higher basal rates of DNA replication than GSTp**^−^** hepatocytes. These characteristics closely reflect those described for GSTp^+^ cells in the intact liver *in vivo*. Thus, this defect in growth regulation persists under culture conditions and therefore appears to be independent of intercellular contacts within the intact organ, and of cytokines, growth factors or hormones circulating in the whole body. The present study shows that considerably more GSTp^+^ cells synthesise and transport *proTGFα* to the nucleus than GSTp**^−^** cells. The nuclear import of this pro-peptide almost always is followed by DNA replication. The enhanced probability of nuclear translocation of *proTGFα* may therefore be essential for the intrinsic growth advantage of the preneoplastic cell population. Compared to the current concepts of signal transduction, nuclear *proTGFα* may not depend on intact erbb-1 receptors on the cell surface. It circumvents the secretion and possible loss of TGF*α* to the outside of the cell and may confer autonomy and an inherent growth advantage, a pathway preferentially used by the preneoplastic cell population.

In an untreated, healthy liver, almost all of the hepatocytes are in the Go-phase of the cell cycle. In the present study, the incidence of nuclei positive for *proTGFα* increased in the course of hepatocarcinogenesis. Since *proTGFα* translocates to the nucleus in the G1-phase of the cell cycle (Grasl-Kraupp *et al*, 2002), the elevated presence of nuclear *proTGFα* in (pre)malignant hepatocytes may be evidence for a G1-status of these cells. Several groups reported that premalignant liver cells show an increased expression of c-myc and cyclin D1, known inducers of the transition from G0 to G1 of the cell cylce (Deguchi and Pitot, 1995; Ramljak *et al*, 2000). On the other hand, the expression of the WAF1/CIP1 gene product, p21 and the c-myc antagonist mad were decreased in hepatocarcinogenesis (Martens *et al*, 1996). Many growth-stimulating factors exert their activity, provided that the target cell is in the G1-phase of the cell cycle. Thus, the enhanced presence of nuclear *proTGFα* in the (pre)neoplastic cell compartment appears to be involved in the overcoming of critical checkpoints of the cell cycle and in a facilitated response of liver (pre)neoplasia towards various growth stimuli.

Transforming growth factor *α* and erbb-1 are upregulated in malignancies of many different organs, including HCC. Novel therapeutic approaches have been focusing on the possible benefit of blocking TGF*α*-evoked signal transduction on the cell surface, for example, by erbb-1 blockade (Levitzki and Gazit, 1995; Mendelsohn, 1997). The present study shows that treatment with mature TGF*α* stimulated DNA synthesis rather in *proTGFα-*neg than in *proTGFα-pos* hepatocytes, which was abrogated by an erbb-1-specific tyrosine kinase inhibitor. In GSTp^+^ hepatocytes, however, mature TGF*α* and tyrphostin exerted no significant effect. Thus, the ‘classical’ signal transduction pathway of mature TGF*α* via erbb-1 seems to be active in the unaltered cell population, while the novel pathway seems to operate preferentially in the (pre)malignant cell compartment. It is therefore tempting to speculate that liver tumours may use alternative pathways for growth stimulation by *proTGFα*. Then, they may be resistant against therapeutic strategies targeted at TGF*α*/erbb-1 interactions on the cell surface.

In conclusion, the present work shows that the incidence of nuclei expressing *proTGFα* is elevated in the course of rat hepatocarcinogenesis, which may reflect and contribute to the inherent growth advantage of (pre)neoplastic hepatocytes. Further research is necessary to elucidate the mechanisms that regulate the different intracellular routes of *proTGFα* and that may link nuclear *proTGFα* to DNA replication.
